# The role of wearable devices and objective gait analysis for the assessment and monitoring of patients with lumbar spinal stenosis: systematic review

**DOI:** 10.1186/s12891-019-2663-4

**Published:** 2019-06-15

**Authors:** Ananya Chakravorty, Ralph J. Mobbs, David B. Anderson, Kaitlin Rooke, Kevin Phan, Nicole Yoong, Monish Maharaj, Wen Jie Choy

**Affiliations:** 1 NeuroSpine Surgery Research Group (NSURG), Sydney, Australia; 2 Neuro Spine Clinic, Suite 7, Level 7. Prince of Wales Private Hospital, Barker Street, Randwick, NSW 2031 Australia; 3 The University of New South Wales (UNSW), Randwick, Australia; 4 Sydney School of Medicine, Sydney University, Sydney, Australia

**Keywords:** Lumbar spinal stenosis, Wearables, Gait assessment, Gait metrics

## Abstract

**Background:**

The purpose of this systematic review was to evaluate the accuracy and reliability of wearable devices for objective gait measurement of Lumbar Spinal Stenosis (LSS) patients, with a focus on relevant gait metrics.

**Methods:**

Systematic searches were conducted of five electronic databases to identify studies that assessed gait metrics by wearable or portable technology. Data was collected according to the Preferred Reporting Items for Systematic Reviews and Meta-Analysis (PRISMA) statement guidelines.

**Results:**

Four articles were identified for inclusion in this review. The objectives, methodology and quality of the studies varied. No single gait metric was investigated in all four studies, making comparison difficult. The most relevant metrics reported included gait cycle, gait velocity, step length and cadence, which were reported in two studies. Two studies explored gait symmetry. Differences between LSS patients and normal healthy subjects are demonstrable using wearable technology.

**Conclusions:**

The measurements of gait cycle, cadence, step length, gait velocity, and number of steps with wearable devices can be used in the gait measurement of LSS patients for initial assessment, and objective outcomes following interventions. However, data and analysis are limited, and further studies are necessary to comment on reliability.

## Background

Lumbar Spinal Stenosis (LSS) is one of the most common indications for spine surgery, and a significant cause of pain and disability in the community. LSS patients classically present with a forward stoop (i.e. trunk flexion) with ambulation, claudicant pain, and paraesthesiae in the buttock, thigh or radiating down into the foot [1]. Decreased walking tolerance and intermittent claudication tends to be relieved by leaning forward [1]. Walking posture often correlates with patients’ symptoms, with back extension exacerbating pain and forward flexion providing variable relief. However, definitive diagnosis is based on the combination of the patient’s history, clinical examination and imaging findings on CT and MRI (CT if not MRI compatible), which need to demonstrate stenosis of the central canal surrounding the cauda equina [2, 3].

Lumbar decompression surgery using standard or minimally invasive techniques [4, 5], with or without fusion, is currently regarded as an appropriate management strategy for LSS when conservative therapies fail [2]. However, it is important to note that although the addition of fusion to lumbar decompression surgery is not associated with a significantly greater improvement than decompression alone, the overall patient quality of life is improved as seen in several studies including the Spine Patient Outcomes Research Trial (SPORT) study [6–8]. While important outcomes such as walking and pain are generally considered favourable with decompression surgery, there are limited tools for assessing these. Current assessments are primarily subjective, such as the Oswestry Disability Index (ODI) and Visual Analogue Scale (VAS), which have limitations. These limitations include: bias of reporting by patient and researcher; no firm consensus on the timing of subjective assessment; absence of continuous assessment to reveal recovery dynamics following an intervention, and the highly subjective nature of patient psychology with individual self-assessment.

The association between gait deterioration and LSS has been long established and explored in the literature. Khodedadah and Eisenstein (1993) established a correlation between gait improvement, particularly gait velocity, in patients with low back pain after lumbar fusion surgery [9]. In recent years, gait analysis has been increasingly utilised for assessing patient outcomes for various other spinal pathologies [10]. Patients with LSS have larger gait variability pre-operatively compared to post-operatively, and when compared to healthy subjects [11, 12]. A range of gait parameters including cadence, gait cycle, step length and step counts have all been proposed to correlate with spinal pathologies [13, 14]. However, traditional gait analysis in a formal laboratory setting is time consuming, equipment and labour-intensive, utilising video analysis, optical motion tracking and analysis, multiple sensors or gyroscopes, and electromyography [3, 15, 16].

Recently, the development and increased availability of wearable technologies for gait analysis has provided a faster and simplified alternative to laboratory-based gait analysis [17–19]. Wearable technologies incorporate aspects of traditional gait analysis techniques such as motion sensors, gyroscopes and accelerometers into everyday wearables such as watches and single piece devices easily attached to the clothing or body of a patient [19]. The incorporation and increased adaptation of these sensors have also decreased the cost for these wearable devices, thereby increasing affordability and facilitating their use in research.

The recent surge of different medical wearables from a range of companies and research facilities, and the reliability, sensitivity and specificity of these different devices poses a potential challenge for accurate medical use and reporting. While still emerging in research, several studies have been carried out to evaluate the usefulness of these wearables for different gait related pathologies. Therefore, the aim of this study is to systematically review the available studies [20] in order to evaluate the accuracy and reliability of different gait parameters (gait cycle, cadence, step length, velocity, step counts and other metrics) for patients with LSS.

## Methods

### Literature analysis

The PRISMA statement guidelines were followed in identifying, screening and selecting studies for inclusion, and extracting data.

### Eligibility criteria

The focus of this review was on journal articles, conference proceedings published in English since the year 2001 that described the use of wearable devices to assess gait quality for patients suffering from lumbar spinal stenosis and neurogenic claudication. The parameters that were analysed in this study include gait cycle, gait velocity, step length, cadence, step count and gait symmetry. Book chapters and review papers were excluded.

### Search strategy and study selection

Relevant studies were initially identified through a systematic search for published papers in the following scientific databases: PubMed (via NLM® database), Medline (via OvidSP), CINAHL (via Ebsco), SportDiscus (via Ebsco) and Google Scholar. The search terms used were “Wearable”, “Gait”, and “Lumbar Spinal Stenosis” (see Table 1).

**Table 1:** Search Strategy

Database	Search strategy	No. of records found	No. of unique abstracts selected	No. of unique articles selected	Articles
PubMed	“gait” AND “wearable”	680	17	0	–
	“gait” AND “spine” AND stenosis	178	4	3	Sun 2018 [3, 21] Lee 2017 [22] Loske 2018 [23]
Medline	“gait” AND “wearable” AND spine	2	1	0	–
	“gait” AND “wearable”	474	15	0	*–*
	“gait” AND “lumbar stenosis”	10	1	0	*–*
CINAHL	“gait” AND “wearable”	209	0	0	*–*
	“gait” AND “stenosis”	102	1	0	*–*
SPORTDiscus	“gait” AND “wearable”	170	3	0	–
	“gait” AND “stenosis”	17	2	0	–
Google Scholar	“gait” AND “wearable” AND “lumbar spinal stenosis”	70	2	0	–
Other (e.g. hand search of citations)		155	6	1	Nagai 2014 [13]
Totals		2067	52	4	

The search of the databases was completed on 1 September 2018 by two authors (AC and RM). Titles and abstracts of all studies identified were screened for relevance. The full text of the record was reviewed if the study appeared relevance or was of uncertain relevance. Studies which were clearly not relevant based on the title and abstract screen were excluded from the review. The full text of all selected relevant records was reviewed, and eligibility was determined using the eligibility criteria defined above.

The quality of each included record was assessed by two authors (AC and RM), and relevant information was extracted. Furthermore, the references of all included studies were hand-searched for additional publications that could be included in this review. At all stages of the study selection process, decisions regarding inclusion or exclusion were made by two authors (AC and RJM). The search was revalidated by a third author (WJC).

### Data extraction

Data was collected with respect to participant characteristics, study design, the sensor type uses and outcomes for each study included in our review. Participant information included number and type of participants, age, and sex. Information about the type, model, sampling frequency and location on the body of each wearable sensor was recorded. The pathology of interest, environment of the study and specific gait parameters were also recorded included walking speed, distance and time. Walking speed was labelled as “self-selected” if participants were walking at a comfortable speed for them individually, but required to maintain the same speed. If participants were allowed to walk at their own speed, it was labelled as “not controlled”.

### Quality assessment

The quality of each of the included articles was assessed using the Standard Quality Assessment Criteria for Evaluating Primary Research Papers from a Variety of Fields (QualSyst tool) [24]. Each article was evaluated by two authors (AC/ RJM) on 13 questions that considered the reporting, external validity, internal validity and power of the study. Each question had three possible answers: “Yes”, “No”, or “N/A.” A Quality Assessment score of less than 0.5 is considered poor quality, and over 0.7 good-excellent [25]. Any discrepancies in scoring between authors were discussed until an agreement was reached.

### Devices

Each study used different wearable devices to measure selected gait metrics. These included:

i. (Nagai et al) Triaxial accelerometers placed on cervical and lumbar spine (WAA-066, ATR Promotions Co., Japan)
ii. (Lee et al) Sensorised smart-shoes (UCLA Wireless Health Institute) using pressure sensors (FSR400, Interlink Electronics, USA)
iii. (Sun et al) Intelligent Device for Energy Expenditure and Activity 3 (MiniSun, LLC, Fresno, CA, USA), accelerometer and gyroscope
iv. (Loske et al) RehaGait System (Hasomed GmbH, Magdeburg, Germany) with accelerometer, gyroscope and magnetometer

## Results

### Search results

**Fig. 1:**
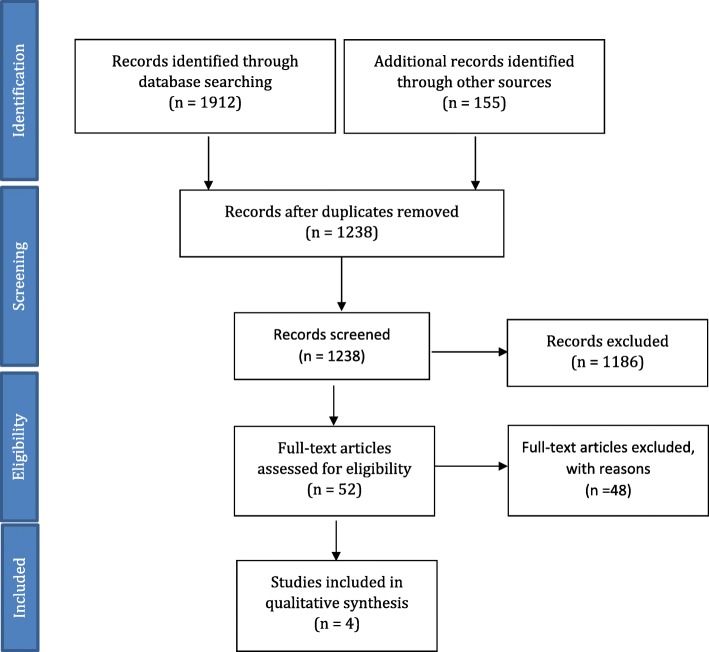
Flow chart outlining the process in this systematic review. Based upon the PRISMA preferred reporting for Systematic reviews [26]

A total of 1912 articles were identified through the database search, and 155 additional articles were identified through a hand search of reference lists. Identifying duplicates, screening of the titles and abstracts led to the removal of 2015 articles, leaving 52 for full-text analysis (Fig. 1). A total of 4 articles met the inclusion criteria (see Table 2) [13, 21–23].

**Table 2:** Summary of articles included for review

Authors	Participant characteristics	Sensor type	Location of sensor	Gait metrics measures	Sampling frequency	Environment	Gait speed	Gait distance/time	Quality assessment score
Nagai et al., 2014 [13]	*N* = 11 (3 female, 8 male) Mean age = 72.8 yrs	Wearable device with triaxial accelerometer (WAA-066, ATR Promotions Co., Japan)	Lumbar spine and cervical spine; 2 sensors)	Postural sway Walking capacity Gait cycle	1 occasion	Indoor horizontal walkway	Self-selected	50 m repetitions until tired (maximum 548 m)	0.54
Lee et al., 2017 [22]	*N* = 15 (11 female, 4 male) Mean age = 58.5 yrs	Smart-shoes (UCLA Wireless Health Institute) with pressure sensors (FSR400, Interlink Electronics, USA)	Insole of shoe (heel, lateral plantar, toe); 5 sensors	Plantar pressure distribution Gait symmetry	1 occasion (preoperative)	Indoor hospital ward	Self-selected	40 m	0.46
Sun et al., 2018 [3, 21]	*N* = 20 (sex not specified) Mean age = 58 yrs	Wearable device with accelerometers, gyroscopes (Intelligent Device for Energy Expenditure and Activity 3; MiniSun, LLC, Fresno, CA, USA)	Chest, thigh ankles and plantar surface of foot; 7 sensors	Gait cycle Cadence Step length Gait velocity	1 occasion	Indoor, well-lit environment	Self-selected	16 m	0.71
Loske et al., 2018 [23]	N = 20 (sex not specified)	Inertial sensors with accelerometer, gyroscope and magnetometer (RehaGait System, Hasomed GmbH, Magdeburg, Germany)	Lateral shoe, lower and upper legs, pelvis; 7 sensors	Gait cycle Gait symmetry Step length Gait velocity Cadence	3 occasions (preoperatively, 10 weeks postop, 12 months postop)	Indoors (clinic)	Self selected	6 min	0.67

### Quality assessment

The results of the quality assessment are outlined in Table 3.

**Table 3:** Quality assessment scores of included studies

Study	Question/objective	Study design	Subject and/or comparison group selection	Subject and/or comparison group characteristics	Random allocation	Blinding of investigators	Blinding of participants	Outcome and exposure measures	Sample size	Analytic measures	Estimate of variance	Confounding	Results	Conclusion	Summary score
Nagai et al. 2014 [13]	Yes	Partial	No	Yes	N/A	N/A	N/A	Partial	Partial	Partial	Yes	No	Partial	Yes	0.54
Lee et al. 2017 [22]	Partial	Partial	Partial	Yes	N/A	N/A	N/A	Partial	Partial	Partial	Yes	No	Partial	Partial	0.46
Sun et al. 2018 [3, 21]	Yes	Yes	Partial	Yes	N/A	No	N/A	Yes	Partial	Partial	Yes	No	Yes	Yes	0.71
Loske et al. 2018 [23]	Yes	Yes	Partial	Yes	N/A	No	N/A	Partial	Partial	Partial	Yes	No	Yes	Yes	0.67

### Gait metrics measured

Gait metrics measured across the studies included gait cycle time, gait symmetry, gait velocity, cadence, step length, number of steps, postural symmetry and plantar pressure distribution [21–23]. No single gait metric was measured in any study.

Gait cycle, gait velocity, step length, and cadence were measured in two of the four studies [21, 23]. Gait cycle was defined as the time between initial contact of one foot with the ground and contact of the same foot, denoting a single stride (synonymous with stride duration). Nagai et al. reported stride frequency in Hertz (i.e. steps per second), which is related to gait cycle [13]. Gait velocity was defined as distance travelled per unit of time in metres per second. Step length was defined as distance between the point of contact of one heel with the point of contact of the alternate heel. Cadence refers to the rate at which a person walks, and was defined as the number of steps per minute. Lee et al. and Loske et al. measured gait symmetry, which was defined as identical gait parameters in each leg, and measured according to the formula *symmetry = (right leg stride time – left leg stride time)/(0.5 x [right leg stride time + left leg stride time]* [22, 23]. Step count, or number of steps, was measured in one study by Sun et al., and was defined as the number of steps taken over 40 m.

The primary metric measured by Lee et al. was pressure distribution on the plantar surface of the foot during gait, describing a complex set of measurements developed by the authors based on multiple plantar pressure sensors. Lack of correlation to other more well-established gait metrics described above makes the significance of this metric uncertain.

Nagai et al. measured postural sway during walking using cervical and lumbar accelerometer sensors [13]. The focus of their study was walking capacity, recording walking difficulty at various distances and whether this was correlated with increased postural sway. However, they did not correlate this with gait symmetry or gait variability or compare these results to age-matched healthy participants. Therefore, whether postural sway per se is an important gait metric cannot be determined.

### Principal gait metrics

Gait cycle, gait velocity, step length and cadence are the most important metrics for patients with lumbar spinal stenosis based on current research. Despite the limited evidence available, these metrics appear to be amenable to wearable/portable gait metric analysis. However, variation in study objectives and methodology make conclusions regarding statistical significance difficult to draw.

i. *Gait Cycle*

Gait cycle was measured in two studies, by Sun et al. and Loske et al.. Sun et al. found a gait cycle of 1.082 ± 0.637 s in healthy volunteers, which was shorter compared to 1.111 ± 0.109 s in patients with LSS. However, a small sample size of only 12 healthy subjects and 20 LSS patients was used, and the authors did not provide statistical comparison between these groups. Loske et al. compared gait cycle preoperatively in 29 LSS patients to 27 healthy subjects, reporting 1.15 ± 0.17 s in LSS patients compared to 1.06 ± 0.08 s in healthy subjects. Loske et al. also compared preoperative gait cycle time to 10 weeks post lumbar decompression, noting a decrease in mean gait cycle to 1.09 ± 0.16 s. Statistical significance was not provided, however both studies indicate that increased gait cycle time in LSS patients can be demonstrated using wearable technology. Nagai et al. reported stride frequency in Hertz (Hz), comparing LSS patients at the beginning of the walking exercise to the end of the walking exercise (1 Hz vs 0.9 Hz). They did not find a statistical difference, and did not compare their values to healthy controls.

ii. *Gait Velocity*

Both Sun et al. and Loske et al. noted decreased gait velocity in LSS patients pre-operatively using their respective wearable devices. Sun et al. report gait velocity of 1.023 ± 0.215 m/second in the LSS cohort preoperatively, compared to 1.158 ± 0.109 m/second in the healthy group, however do not provide further comparison postoperatively. Loske et al. show a similarly decreased gait velocity in LSS patients preoperatively of 1.09 ± 0.34 m/second, compared to 1.29 ± 0.27 m/second in healthy controls. They also reported an increase in gait velocity at 10 weeks postoperatively to 1.16 ± 0.28 m/second.

iii. *Step Length*

Sun et al. reported a decreased step length in LSS patients compared to health controls (0.557 ± 0.085 m versus 0.625 ± 0.513 m). Loske et al. report decreased described stride length rather than step length (which consists of two step lengths, right and left combined), and reported decreased stride length LSS patients preoperatively (1.18 ± 0.26 m) compared to healthy controls (1.34 ± 0.26 m). They reported some improvement at 10 weeks postoperatively (1.21 ± 0.20 m), however this was not marked.

iv. *Cadence*

The two studies that measured cadence found conflicting results with respected to cadence in LSS patients compared to healthy controls. Sun et al. report cadence of 107.626 ± 7.102 steps/min in healthy subjects, compared to 109.219 ± 10.499 steps/min in LSS patients. On the contrast, Loske et al. report cadence of 106.6 ± 14.7 steps/min in preoperative LSS patients compared to 113 ± 12.7 steps/min in healthy subjects, increasing to 111.6 ± 13.5 steps/min at 10 weeks postoperatively.

v. *Step count*

Of the three studies, only Sun et al. measured step count. They reported mean step count of 94.000 ± 36.245 in LSS patients over a distance of 16 m, however did not provide data for healthy subjects. In terms of total daily step counts however, this data is still lacking.

vi. *Gait symmetry*

Lee et al. and Loske et al. examined gait symmetry in LSS patients. Lee et al. stated that gait symmetry was recorded in all patients, however did not directly report the results of gait symmetry in their cohort. They stated that there was no correlation between gait symmetry and their outcome of interest, the ODI, but found statistically significant correlation when gait symmetry was combined with other measurements of pressure distribution. This data is difficult to interpret in isolation. Loske et al. found greater gait asymmetry in LSS patients in all phases of the gate cycle, which persisted at 10 weeks follow up but had normalised by 12 months.

## Discussion

LSS is a disabling condition with significant economic, physical and psychological cost. It represents the most common indication for spine surgery in people older than 65 years. Most studies regarding LSS and outcomes of surgery are based on patient-reported information which may be subjective, inaccurate or incomplete [27, 28]. There is little objective data on functional changes following surgical intervention for LSS. The use of accelerometers to evaluate activity post spinal surgery is a promising avenue to provide objective measurements as compared to self-completed questionnaires or formal laboratory-based gait assessment [29]. Based on the limited data available from the 4 identified studies, we conclude that the measurements of gait velocity, cadence, step length, number of steps and gait symmetry are useful in the assessment of decline and recovery in patients with LSS. The small number of studies and variation in methodology used indicate that further studies investigating the capacity of wearable gait metric measurement to provide reliable results are necessary.

Normal gait speed of adults and LSS patients has been studied by Bohannon et al. and Conrad et al. respectively [30, 31]. Comfortable gait speed for adults aged 20–79 has been reported to be a mean of 1.33–1.46 m/s for men and 1.27–1.41 m/s for women [30]. LSS patients have been found to have generally slower gait speed, with Conrad reporting figures of 1.01 ± 0.33 m/s for men and 0.75 ± 0.24 m/s for women [31]. The two studies that measured gait velocity in the current review demonstrated decreased gait velocity in LSS patients. Step and stride length was also identified as a significant metric for the LSS patient, and have been shown to be reduced in LSS patients in the available studies with the time frame for postoperative improvement uncertain in the LSS patient. Our review also found that the two wearable systems used by Sun et al. and Loske et al. demonstrated increased gait cycle in LSS patients compared to normal controls.

In general, the mean step length and velocity in LSS patients is less than for normal adults, which is likely to reflect pain or neurological deficit in these patients. The shorter step length and slower velocity may also represent compensation to maintain stability and reduce the falls risk. Data comparing preoperative and postoperative gait metrics in LSS patients in the same study cohort would be clinically helpful for surgeons to provide objective outcome information that could be correlated against subjective symptomatic and functional outcomes. Only one study (Loske et al) compared preoperative and postoperative measurements in their gait analysis, which speaks to the need for more and better quality data to aid clinical practice.

There are limitations in this systematic review. The five primary gait parameters examined in this study are essential to clinically evaluate patients’ gait. Although more gait parameters, such as gait phase and joint angle may be analysed for improved assessment of patients’ gait, the authors believe that a simple (single point fixation) wearable provides adequate assessment of decline and recovery for the LSS patient. Further studies to assess the reliability of different wearable technologies for gait analysis are necessary. However preliminary evidence and pilot studies suggest that simple wearables are as effective and more convenient in providing standard gait metrics compared to traditional in-laboratory gait analysis.

Although the current available studies that uses gait parameters as an evaluation tool for LSS is sparse, our findings indicate the above parameters are suitable to be used to aid monitoring surgical and treatment outcomes. The recent incorporation and increased availability of wearable measurement devices into daily gadgets such as smartphones and smartwatches ease the process of data collection for these parameters even expanding to an outside-laboratory setting. With the advancement of such devices, a larger volume of data regarding patient activity can be collected and analysed, and may even be translated into future clinical assessment to assist in evaluating patients’ outcome.

## Conclusion

Although the data on gait metrics from wearable devices for LSS patients is limited, the available evidence suggests that this group exhibits decreased step length and gait velocity compared to normal subjects. Helpful gait metrics are gait cycle, step length, velocity and number of steps, which have the potential to be easily measured using new wearable devices for preoperative and postoperative assessment of LSS patients.

## Data Availability

Not applicable, as no data was used. The EndNote reference library used in this study can be provided via email if required by the BMC journal (please contact last author WJC). The search strategies used in this systematic review have been included and are reproducible on public databases.

## Abbreviations

LSS: Lumbar Spinal Stenosis
ODI: Oswestry Disability Index
VAS: Visual Analogue Scale
